# The Use of Arthroscopy in Diagnosis and Operative Treatment of Knee Posterolateral Corner Injuries: A Narrative Review

**DOI:** 10.3390/jcm15135257

**Published:** 2026-07-06

**Authors:** Claudio Domenico Cobisi, Fortunato Giustra, Alessandro Carrozzo, Giuseppe Rovere, Carmelo Burgio, Lawrence Camarda, Marcello Capella, Francesco Bosco

**Affiliations:** 1 Department of Precision Medicine in the Medical, Surgical and Critical Care Area (ME.PRE.C.C.), University of Palermo, 90133 Palermo, Italy; claudiodomenico.cobisi@community.unipa.it (C.D.C.); lawrence.camarda@unipa.it (L.C.); 2 Department of Orthopedics and Traumatology, G.F. Ingrassia Hospital Unit, ASP 6, 90131 Palermo, Italy; 3 Ortopedia e Traumatologia 2, Ospedale San Giovanni Bosco, Azienda Sanitaria Locale Città di Torino, Piazza Donatori del Sangue 3, 10154 Turin, Italy; fortunato.giustra@aslcittaditorino.it; 4 Link Campus University, 00165 Rome, Italy; a.carrozzo@unilink.it; 5 Department of Orthopaedics and Traumatology, Policlinico Tor Vergata Foundation, 00133 Rome, Italy; rovere292@hotmail.com; 6 Adult Reconstruction and Joint Replacement Service, Department of Orthopaedic Surgery, Hospital for Special Surgery, New York, NY 10021, USA; burgioc@hss.edu; 7 Department of Orthopaedic Surgery, Centro Traumatologico Ortopedico (CTO), Via Gianfranco Zuretti, 29, 10126 Turin, Italy; marcello.capella84@gmail.com

**Keywords:** knee surgery, posterolateral corner, multiligament injuries, arthroscopy

## Abstract

Injuries to the posterolateral corner (PLC) of the knee represent a complex clinical entity that is frequently underdiagnosed and undertreated, often leading to persistent instability and failure of concomitant ligament reconstructions. Although open surgical approaches to the PLC have been extensively described, the role of arthroscopy in the diagnosis and management of these injuries remains less clearly defined despite increasingly encouraging clinical and biomechanical results. The aim of this narrative review was to analyze the role of knee arthroscopy in the diagnosis, evaluation, and treatment of PLC injuries. A comprehensive literature review was performed focusing on studies investigating arthroscopic findings, diagnostic signs, and arthroscopic or arthroscopy-assisted techniques for PLC management. Relevant clinical, anatomical, biomechanical, and surgical studies were analyzed to provide an integrated arthroscopy-oriented perspective. Arthroscopy enables direct visualization of key posterolateral structures and identification of characteristic diagnostic findings, such as the lateral drive-through sign, potentially improving detection of subtle or combined PLC injuries. In addition, arthroscopic assessment facilitates evaluation of associated intra-articular lesions and may contribute to more accurate characterization of injury patterns. Emerging arthroscopic and arthroscopy-assisted reconstruction techniques may offer advantages in selected cases by supporting tailored surgical strategies, accurate graft positioning, and reduced surgical morbidity. Knee arthroscopy is assuming an increasingly important role in the comprehensive management of PLC injuries, extending beyond the treatment of associated intra-articular pathology alone. Integration of arthroscopy into the diagnostic and therapeutic algorithm of PLC injuries may improve surgical decision-making and patient-specific management. Nevertheless, further high-quality clinical studies are required to establish standardized arthroscopic criteria and validate the long-term clinical advantages of arthroscopy-guided approaches.

## 1. Introduction

Knee posterolateral corner (PLC) injuries represent a complex and frequently underdiagnosed condition that plays a critical role in knee stability by resisting varus stress, external tibial rotation, and posterior tibial translation [1]. The PLC consists of multiple static and dynamic stabilizers, including the lateral collateral ligament (LCL), popliteus tendon (PT), popliteofibular ligament (PFL), and several capsuloligamentous structures, which together contribute to the intricate anatomy and biomechanics of the posterolateral knee compartment [2,3].

Although historically considered uncommon, growing evidence suggests that PLC injuries occur more frequently than previously recognized, largely owing to improved diagnostic awareness and advances in imaging techniques [4,5,6]. Epidemiological studies have reported PLC involvement in up to 16% of acute ligamentous knee injuries and approximately 9% of acute knee injuries associated with hemarthrosis [4,5,6]. Importantly, isolated PLC lesions are relatively rare, whereas most injuries occur in association with anterior or posterior cruciate ligament (ACL/PCL) tears or multiligament knee injuries [4,5,6].

Failure to recognize and adequately treat PLC injuries has been associated with persistent instability, altered knee biomechanics, and inferior outcomes following cruciate ligament reconstruction [7,8,9]. Consequently, accurate diagnosis and appropriate management are essential to restore joint stability and optimize long-term functional outcomes.

Current evidence and expert consensus suggest that low-grade PLC injuries may be managed conservatively, whereas high-grade, chronic, or combined lesions generally require surgical treatment [3,4,7,8,10,11]. This principle is consistent with evidence from the ligament injury literature, where persistent residual laxity has been associated with inferior patient-reported outcomes and has been shown to support surgical reconstruction over conservative management in appropriately selected patients [12]. In this setting, anatomic reconstruction techniques have progressively emerged as the preferred surgical strategy because of their superior ability to restore native knee biomechanics and reduce failure rates compared with non-anatomic procedures, although these operations remain technically demanding [8,9,13].

More recently, minimally invasive and arthroscopic-assisted techniques have gained increasing attention as potential alternatives to traditional open approaches [13,14,15]. Arthroscopy may improve visualization of the posterolateral compartment, facilitate identification of subtle instability patterns and associated intra-articular lesions, and reduce surgical morbidity [13,14,16]. An additional advantage of arthroscopy is the possibility of simultaneously diagnosing and treating associated intra-articular pathology, including meniscal tears, chondral lesions, and cruciate ligament injuries, within the same surgical procedure [14,17,18]. Nevertheless, despite encouraging preliminary results, the current evidence supporting arthroscopic PLC surgery remains limited and is predominantly based on technical notes, cadaveric investigations, and small case series [14,15,17,19,20,21,22,23,24,25,26,27].

The aim of this narrative review is to critically analyze the current evidence on the role of arthroscopy in the diagnosis and management of knee PLC injuries, with particular emphasis on arthroscopic diagnostic findings, intraoperative assessment, surgical techniques, and their potential impact on treatment strategies and clinical decision-making.

## 2. Materials and Methods

This study was designed as a narrative review aimed at providing a comprehensive and clinically oriented overview of the current evidence regarding the role of arthroscopy in the diagnosis and treatment of knee PLC injuries.

A structured literature search was performed using the PubMed, Web of Science, Scopus, and Embase databases to identify relevant studies published up to February 2026. The search strategy combined Medical Subject Headings (MeSH), where applicable, and free-text terms related to posterolateral corner injuries and arthroscopic management. The following keywords were combined using Boolean operators: (“posterolateral corner” OR “PLC” OR “posterolateral instability” OR “posterolateral rotatory instability”) AND (“arthroscopy” OR “arthroscopic reconstruction” OR “arthroscopy-assisted reconstruction” OR “popliteus” OR “popliteofibular ligament” OR “lateral collateral ligament”). The search strategy was adapted as appropriate for each database. The reference lists of all relevant articles were manually screened to identify additional eligible studies.

Studies were selected according to their relevance to the objectives of this review, with particular emphasis on anatomical, biomechanical, diagnostic, technical, and clinical aspects of arthroscopic management of PLC injuries. Clinical studies, cadaveric investigations, biomechanical studies, technical notes, case reports, and relevant narrative or systematic reviews were considered. Preference was given to studies with greater methodological quality, clinical relevance, and consistency with current concepts of PLC anatomy, biomechanics, diagnosis, and surgical treatment.

The selected literature was reviewed qualitatively to provide an integrated overview of the current role of arthroscopy in the diagnosis and treatment of PLC injuries. Owing to the narrative design of the review and the heterogeneity of the available evidence, no formal risk-of-bias assessment or quantitative synthesis was performed.

## 3. Results

### 3.1. Anatomy, Biomechanics and Historical Issues

The PLC of the knee represents a complex anatomical and biomechanical region composed of multiple static and dynamic stabilizers that play a fundamental role in maintaining lateral, rotational, and posterior knee stability. The primary stabilizing structures include the LCL, PT, and PFL, which act synergistically to resist varus stress, external tibial rotation, and posterior tibial translation [1,2,19].

**Figure 1:**
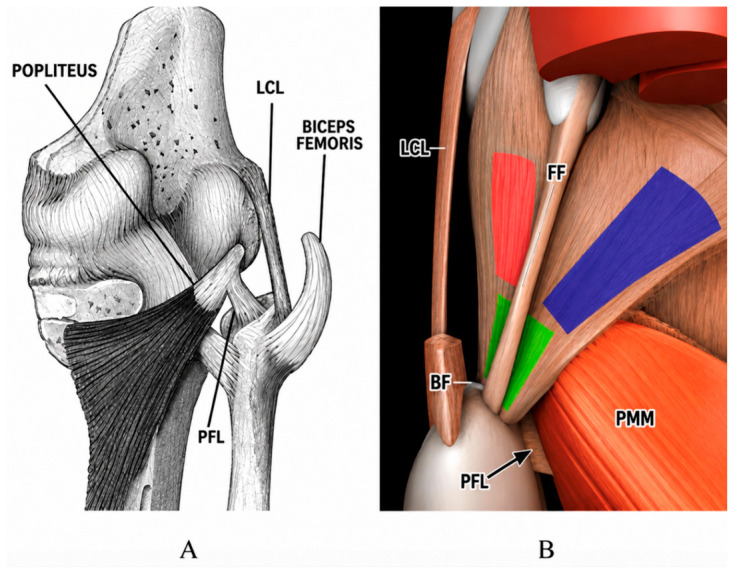
Knee posterolateral corner anatomy. (**A**) Anatomical illustration of the posterolateral corner of the knee showing the lateral collateral ligament (LCL), popliteus tendon, popliteofibular ligament (PFL), and biceps femoris tendon. (**B**) Three-dimensional anatomical representation of the posterolateral corner illustrating the lateral collateral ligament (LCL), fabellofibular ligament (FF), biceps femoris (BF), popliteofibular ligament (PFL), and popliteus muscle (PMM). Reproduced from Agha et al. [31] under the Creative Commons CC BY-NC-ND 4.0 licence.

The LCL originates from the lateral femoral epicondyle and inserts onto the fibular head, functioning as the primary restraint to varus loading, particularly between 20° and 30° of knee flexion [3,28,29]. The PT arises from the lateral femoral condyle and inserts onto the posterior tibia, serving as a dynamic stabilizer that controls external tibial rotation, especially during the early phases of knee flexion [29,30]. The PFL extends from the musculotendinous junction of the popliteus to the fibular head and acts as a critical stabilizer against external rotation and posterior tibial translation, particularly in synergy with the PCL [1,3] (Figure 1).

In addition to these primary stabilizers, several secondary structures contribute to posterolateral stability, including the lateral capsule, arcuate ligament complex, fabellofibular ligament, biceps femoris tendon, and iliotibial band [2,6]. Although often considered secondary restraints, these structures become increasingly relevant near full extension, where capsular constraints significantly contribute to rotational and varus stability.

From a biomechanical perspective, the PLC functions as a key restraint against complex multiplanar loads. Cadaveric sectioning studies have demonstrated that isolated LCL injury primarily increases varus laxity, whereas combined lesions involving the popliteus complex and PFL result in substantial increases in external tibial rotation and posterolateral rotatory instability [3,7]. Importantly, the PLC also demonstrates a close biomechanical interaction with the cruciate ligaments. PLC deficiency has been shown to increase forces transmitted through both anterior and posterior cruciate ligament grafts, particularly under rotational loading conditions, thereby predisposing reconstructed knees to graft overload and failure [4,8].

This biomechanical interplay translates into clinically heterogeneous instability patterns, especially in multiligament knee injuries, where unrecognized PLC deficiency may compromise overall joint stability and negatively affect the outcomes of cruciate ligament reconstruction. Consequently, accurate characterization of PLC injury patterns is essential to guide treatment selection and surgical planning.

Among available classification systems, the Fanelli and Larson classification remains one of the most widely adopted frameworks for categorizing PLC injuries by degree of rotational and varus instability [6,10]. Type A lesions involve isolated injury to the popliteus complex and are characterized by increased external tibial rotation without significant varus laxity. Type B lesions include additional involvement of the LCL, resulting in combined rotational and mild varus instability, whereas Type C lesions represent complete PLC disruption, frequently associated with cruciate ligament injuries and severe multidirectional instability [6,10].

Current evidence suggests that low-grade, isolated PLC injuries may be managed conservatively, whereas high-grade, chronic, or combined lesions generally require surgical reconstruction due to persistent instability and altered knee biomechanics [2,5,11]. Nevertheless, treatment decisions should not rely exclusively on injury grading, as even subtle PLC insufficiency may compromise cruciate ligament reconstructions and therefore require surgical management in selected combined injury patterns [2,5,11].

### 3.2. Limitations of Traditional Open Techniques and Rationale for Arthroscopic Approaches

Historically, open repair techniques for PLC injuries have shown high failure rates, with several studies reporting rates of 37% to 40%, significantly higher than those observed after reconstruction procedures (approximately 6% to 9%) [32,33]. These unsatisfactory outcomes have mainly been attributed to the complex anatomy and biomechanics of the PLC, which are difficult to restore using non-anatomic repair techniques. Furthermore, limited exposure and visualization with traditional open approaches may contribute to underdiagnosis of associated ligamentous and intra-articular lesions, a well-recognized cause of persistent instability and cruciate ligament graft failure [11,32]. In response to these limitations, a progressive shift toward anatomic reconstruction techniques and minimally invasive approaches has emerged.

Arthroscopy provides improved visualization of intra-articular and posterolateral structures, allowing identification of subtle PLC-related instability patterns and more accurate intraoperative assessment. It may also improve recognition of combined lesions that are often underestimated on clinical examination and imaging. Recent comparative studies have shown that arthroscopic and arthroscopy-assisted techniques may provide clinical outcomes comparable to open reconstruction, while offering potential advantages, including reduced surgical morbidity, improved postoperative recovery, and enhanced visualization, which supports their diagnostic role in PLC pathology [6,13,16].

### 3.3. Diagnostic Role of Arthroscopy

**Figure 2:**
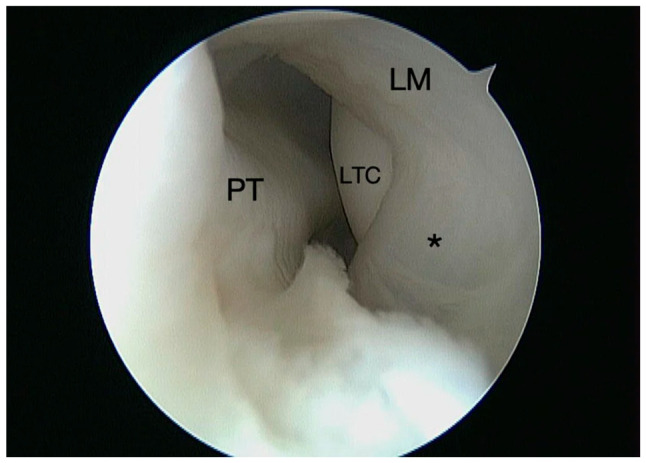
Arthroscopic lateral gutter view of the knee showing the popliteus tendon (PT), lateral meniscus (LM), and lateral tibial condyle/cartilage (LTC). The asterisk (*) indicates widening of the lateral compartment associated with posterolateral rotatory instability (“lateral drive-through sign”). Reproduced from Zdanowicz et al. [36] under the Creative Commons CC BY-NC-SA 3.0 licence.

Among arthroscopic diagnostic findings, the lateral drive-through sign is the most widely described and reflects increased laxity of the lateral compartment in the setting of posterolateral rotatory instability [34]. Feng et al. clarified its biomechanical basis, demonstrating that it reflects combined injury patterns rather than isolated injury of a single structure. Although the lateral drive-through sign is widely recognized as a useful arthroscopic indicator of PLC insufficiency, robust diagnostic accuracy studies reporting sensitivity, specificity, and confidence intervals are currently lacking. Therefore, it should be interpreted as an adjunctive intraoperative finding and correlated with clinical examination and imaging findings. Clinically, this sign may reveal subtle or previously unrecognized PLC injuries, particularly in the setting of multiligament knee instability [2,35] (Figure 2).

Although magnetic resonance imaging (MRI) remains the first-line imaging modality for evaluating PLC injuries, subtle, partial, and combined lesions may still be underestimated, particularly when secondary stabilizers are involved or standard imaging protocols are used. Several studies [2,7,29] have reported discrepancies between preoperative MRI findings and intraoperative assessment, highlighting that arthroscopy may reveal clinically relevant instability patterns and associated lesions that are not always apparent on static imaging. Therefore, arthroscopy should be considered complementary to MRI, particularly in patients with persistent clinical suspicion despite inconclusive imaging findings [7,29].

Arthroscopy also enables detailed evaluation of key anatomical regions, including the popliteus hiatus and posterolateral recess, while supporting the planning of arthroscopy-assisted reconstruction techniques. Furthermore, it allows reliable identification of critical anatomical landmarks, such as the PT, fibular head region, and surrounding neurovascular structures, thereby improving surgical orientation and procedural safety [14].

### 3.4. Arthroscopic Surgical Techniques

The main arthroscopic techniques currently described in the literature are summarized below, highlighting indications, reconstructive concepts, key surgical steps, advantages, and limitations to provide an overview of the most reported procedures and their clinical applications (Table 1). Given the narrative nature of this review, the accompanying figures are intended to illustrate the final graft or repair construct of each technique, thereby facilitating comparison of the different reconstructive concepts and anatomical restoration strategies. Therefore, the figures focus on the final configuration of the reconstruction rather than providing a step-by-step depiction of the surgical procedure.

#### 3.4.1. Anatomical Reconstruction Procedures

##### Selective Popliteus Tendon Reconstruction (Feng et al. [20])

**Figure 3:**
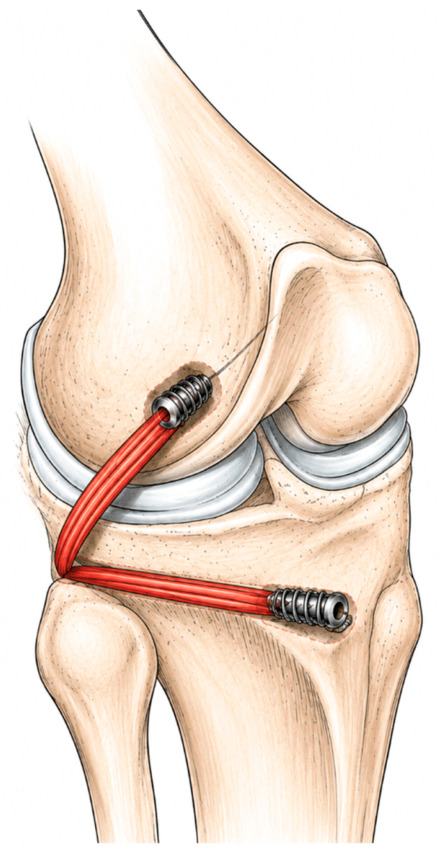
Schematic illustration of the final graft configuration in the all-arthroscopic selective popliteus tendon (PT) reconstruction described by Feng et al. [20].

Feng et al. described an all-arthroscopic sling-type reconstruction designed to restore dynamic popliteus tendon function in patients with isolated posterolateral rotatory instability (Fanelli and Larson Type A lesions) [20]. The procedure is performed through standard anterior portals combined with posteromedial, posterolateral, and trans-septal portals, allowing direct visualization of the PCL and identification of the popliteus musculotendinous junction. After exposure of the posterior tibia, a tibial tunnel is created from the region of Gerdy’s tubercle to the popliteus musculotendinous junction, reproducing the native course of the tendon [20]. On the femoral side, a socket is prepared at the anatomical popliteus footprint under arthroscopic guidance. A semitendinosus autograft or tibialis anterior allograft is then passed through the tibial tunnel and advanced into the femoral socket, creating a sling construct that reproduces the dynamic stabilizing function of the native PT. The graft is fixed using bioabsorbable interference screws after appropriate tensioning with the knee flexed and the tibia maintained in neutral rotation (Figure 3).

The reported advantages include precise arthroscopic identification of the popliteus musculotendinous junction and femoral insertion, structures that are often difficult to locate accurately during open procedures [20]. Furthermore, the technique minimizes soft-tissue dissection, avoids extensive posterolateral exposure and capsulotomy, reduces the risk of iatrogenic neurovascular injury, and may be combined with concomitant arthroscopic posterior cruciate ligament reconstruction when required. However, the procedure reconstructs only the PT and does not directly address the popliteofibular ligament, limiting its applicability in cases of combined or high-grade posterolateral corner deficiency. In their initial clinical series of six patients, the authors reported restoration of external tibial rotational stability comparable to that achieved with traditional open reconstructions [20].

##### Arthroscopic Popliteofibular Ligament Reconstruction of the Knee (Song et al. [21])

This technique is primarily indicated for patients with posterolateral rotatory instability related to PFL insufficiency, particularly in the absence of severe varus laxity [21].

**Figure 4:**
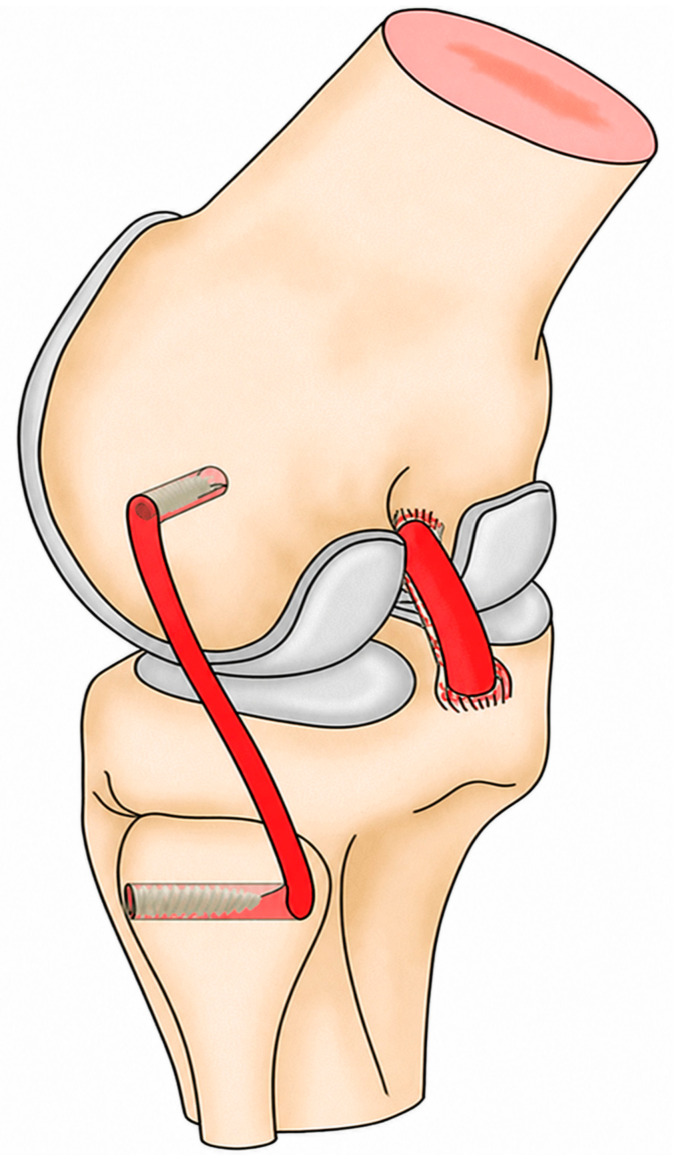
Schematic illustration of the final graft configuration in the all-arthroscopic popliteofibular ligament (PFL) reconstruction described by Song et al. [21].

The procedure aims to restore rotational stability by anatomically reconstructing the PFL using an all-arthroscopic approach. Standard anterior portals are established, followed by posterolateral visualization to identify the PT and fibular head region using an accessory lateral gutter portal. After preparation of the PFL area, a tunnel is created in the fibular head using an ACL guide, and a graft, typically a hamstring tendon or tibialis anterior allograft, is passed from the popliteus musculotendinous junction to the fibula to reproduce the native course of the PFL [37]. Fixation is performed at the fibula with an interference screw at 30° of knee flexion and at the femoral socket with a bioabsorbable screw under arthroscopic visualization (Figure 4).

This technique was reported in a young active male patient and demonstrated satisfactory clinical results at 2-year follow-up. The reported advantages are similar to those described by Feng et al., including improved visualization and reduced surgical morbidity, and the technique is mainly indicated for selected low-grade PLC injuries [20,21].

##### Arthroscopic-Assisted Anatomical Reconstruction of the PLC (Ahn et al. [17])

The arthroscopic-assisted technique described by Ahn et al. aims to achieve anatomical reconstruction of the PLC by restoring the LCL and PFL complex through a combined arthroscopic and mini-open approach [17]. The procedure begins with standard diagnostic arthroscopy, followed by posterolateral visualization to identify key anatomical landmarks, including the popliteus hiatus and lateral femoral condyle. Arthroscopy is used to guide the localization of the femoral footprints and assess associated intra-articular pathology.

**Figure 5:**
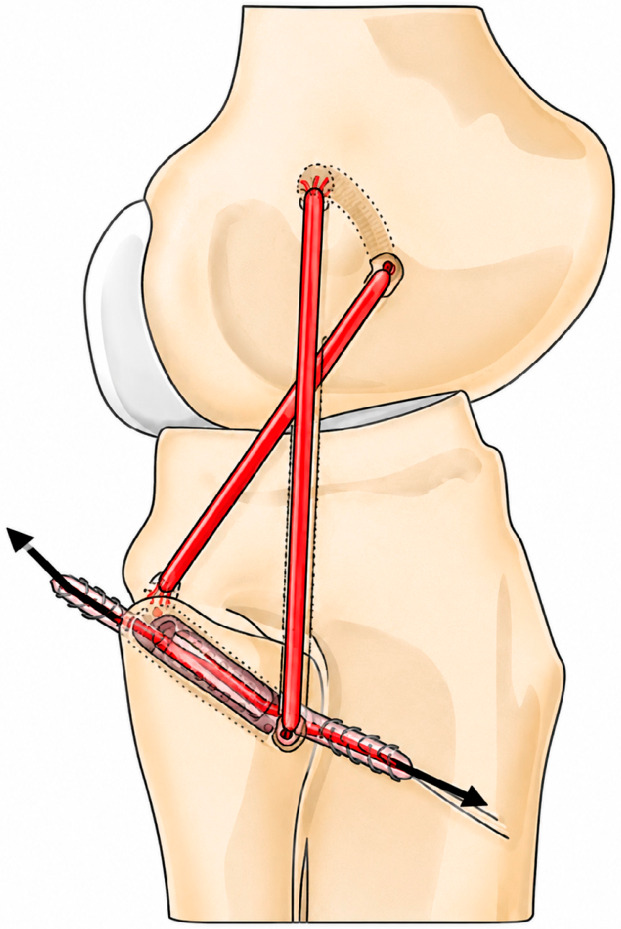
Schematic illustration of the final graft configuration in the arthroscopy-assisted anatomical posterolateral corner (PLC) reconstruction described by Ahn et al. [17]. Black arrows indicate the direction of graft passage and final tensioning.

A limited lateral approach is subsequently performed to expose the fibular head, allowing safe preparation of a fibular tunnel while protecting the common peroneal nerve. Femoral tunnels are created at the anatomical insertions of the LCL and PT, and a tendon graft is passed through the fibular tunnel and fixed proximally to reproduce the native orientation of the reconstructed structures. Arthroscopic guidance is used throughout the procedure to confirm tunnel positioning, graft trajectory, and restoration of lateral compartment stability (Figure 5).

This technique combines the accuracy of anatomical reconstruction with the advantages of arthroscopic visualization while reducing the extent of soft-tissue dissection compared with traditional open approaches [17]. It directly builds upon the anatomical principles established by LaPrade and Arciero [19,22,23], representing an evolution toward less invasive procedures while maintaining the goal of restoring both varus and rotational stability through anatomic multiligament reconstruction. Therefore, it is mainly indicated for high-grade PLC injuries (Fanelli and Larson Type B/C) [19,22,23].

##### All-Arthroscopic Technique for Complex Posterolateral Corner Reconstruction (Frings et al. [14])

Arthroscopic visualization of the posterolateral corner requires a systematic portal strategy, typically including high anterolateral (AL), anteromedial (AM), posteromedial (PM), and posterolateral (PL) portals, combined with a transseptal approach. The procedure begins with standard diagnostic arthroscopy through the anterior portals, followed by establishment of the posteromedial portal under direct visualization, ensuring safe placement and protection of the saphenous nerve [14].

The posteromedial portal is created by advancing the arthroscope through the intercondylar notch beneath the posterior cruciate ligament, allowing access to the posteromedial recess. Accurate positioning of this portal is critical, as it facilitates both visualization and instrumentation of the posterior compartments and serves as the working portal for subsequent steps.

**Figure 6:**
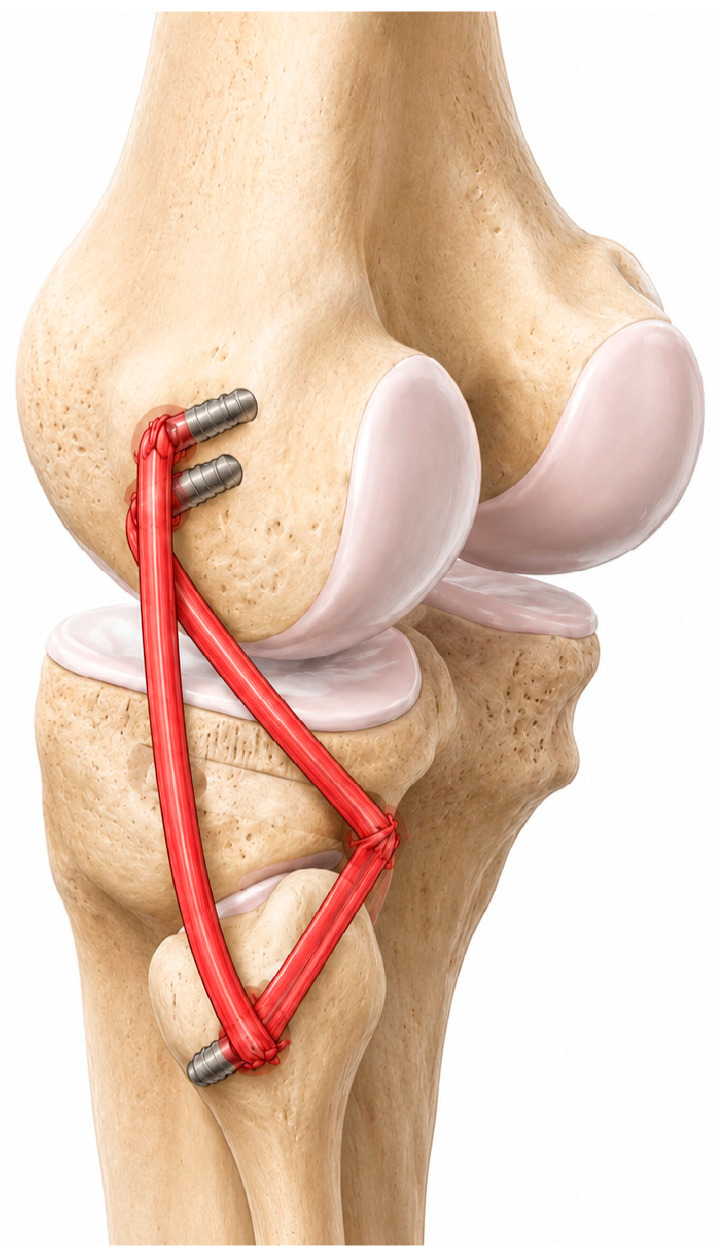
Schematic illustration of the final graft configuration for the all-arthroscopic multiligament posterolateral corner (PLC) reconstruction described by Frings et al. [14].

The transseptal approach is then developed to enable communication between the posteromedial and posterolateral compartments, allowing comprehensive visualization of the posterolateral structures [14]. Under direct arthroscopic visualization, the popliteal sulcus (PS), fibular head (FH), and femoral insertion sites of the lateral collateral ligament LCL and PT are identified. A tibial tunnel is created from the anterior tibia toward the PS, followed by preparation of a transfibular tunnel reproducing the insertion of the PT and LCL. Separate femoral tunnels are then drilled at the anatomic footprints of the LCL and PT. Hamstring autografts are subsequently passed through the tibial, fibular, and femoral tunnels to reconstruct the PT, PFL, and LCL in a fibula-based configuration. Final fixation is typically performed with interference screws or cortical fixation devices while maintaining the knee in slight flexion and neutral rotation to optimize graft tensioning (Figure 6).

In cases of limited access, such as revision surgery or significant scarring, alternative approaches, including a transcondylar intercruciate pathway, may be required to optimize exposure [14].

##### All-Arthroscopic Reconstruction of PLC (Liu et al. [15])

The all-arthroscopic anatomic reconstruction described by Liu et al. is designed to restore the primary stabilizing structures of the PLC, including the LCL, PT, and PFL, using a fully arthroscopic approach in a cadaveric model [15].

The procedure begins with the establishment of standard anterior portals, followed by the creation of posteromedial and posterolateral portals to allow access to the posterior compartments. A transseptal approach is then developed to enable communication between the posteromedial and posterolateral recesses, facilitating comprehensive visualization of the PLC. Through this approach, key anatomical landmarks are identified, including the PT, fibular head, and lateral femoral condyle.

After adequate exposure, the fibular head is prepared arthroscopically, and a fibular tunnel is created to reproduce the distal insertions of the posterolateral structures. Subsequently, femoral tunnels are drilled at the anatomical footprints of the LCL and PT under arthroscopic guidance, ensuring accurate positioning relative to intra-articular landmarks.

A tendon graft, typically a hamstring autograft, is then introduced and passed through the fibular tunnel, with its limbs directed toward the femoral tunnels to recreate the anatomical orientation of the LCL and popliteus complex. The graft configuration allows simultaneous reconstruction of both static and dynamic stabilizers of the PLC. Fixation is performed under controlled tensioning conditions, typically with the knee in slight flexion and neutral rotation, to restore physiological joint alignment.

**Figure 7:**
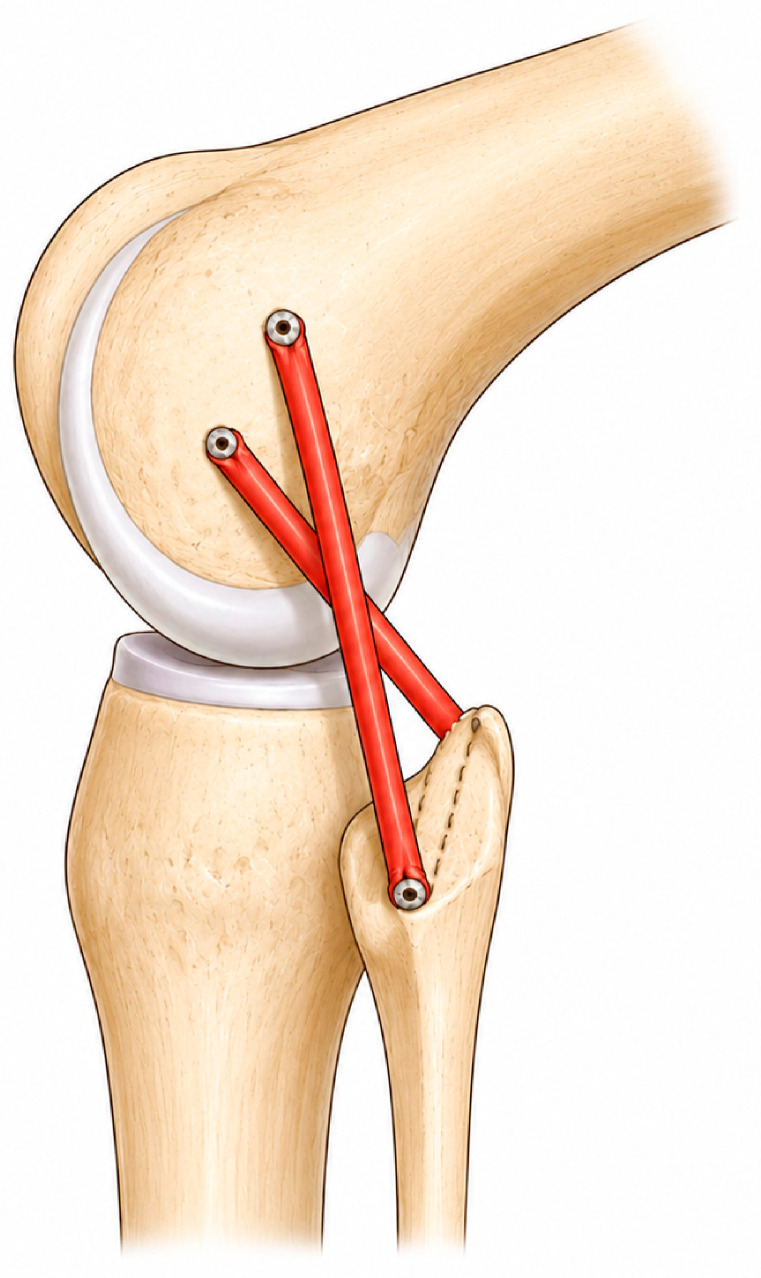
Schematic illustration of the final graft configuration in the all-arthroscopic anatomical posterolateral corner (PLC) reconstruction described by Liu et al. [15].

Arthroscopic reassessment is performed to confirm appropriate graft positioning and restoration of lateral compartment stability (Figure 7). In their biomechanical evaluation, Liu et al. demonstrated that this all-arthroscopic anatomic reconstruction could restore near-normal varus and rotational stability in a cadaveric model, supporting the feasibility of fully arthroscopic PLC reconstruction [15].

##### All-Arthroscopic Stabilization of the Posterolateral Joint Capsule (Ohnishi et al. [24])

The arthroscopic technique described by Ohnishi et al. focuses on direct visualization and management of the PT through a posterior compartment approach [24]. Using standard anterior portals combined with posteromedial and posterolateral access, often facilitated by a transseptal technique, the procedure allows identification of key anatomical landmarks, including the popliteus hiatus and its femoral insertion. After adequate exposure, the PT can be repaired or reconstructed under arthroscopic guidance, typically through preparation of the femoral footprint and fixation using suture anchors or graft-based techniques.

This approach is primarily intended to address lesions involving the PT and may be considered in selected cases of posterolateral rotatory instability associated with focal dysfunction of this structure. The minimally invasive nature of the technique enables simultaneous evaluation and treatment of associated intra-articular pathology while preserving soft tissues.

However, considering the complex multistructural anatomy of the PLC, this technique does not aim to restore all stabilizing components and is mainly indicated for patients with rotational instability (Type B) without significant involvement of the fibular collateral ligament, PFL, or other major posterolateral stabilizers [24].

#### 3.4.2. Arthroscopic Repair Procedures

##### All-Arthroscopic Repair of Arcuate Avulsion Fracture (Zhang et al. [25])

The technique described by Zhang et al. was developed for the treatment of arcuate complex avulsion fractures and aims to restore PLC integrity through minimally invasive fixation [25].

The procedure begins with standard diagnostic arthroscopy through anterolateral and anteromedial portals, allowing assessment of the lateral compartment and identification of associated intra-articular lesions. Additional posteromedial and posterolateral portals may be created to improve visualization of the posterolateral recess and fibular head region.

After exposure, the avulsed fragment at the fibular head is identified and mobilized. Careful debridement of the fracture bed is performed to promote healing while preserving soft-tissue attachments, including the LCL and PFL complex. Arthroscopic visualization allows accurate assessment of fragment size, displacement, and associated soft-tissue injury.

Reduction of the avulsed fragment is achieved under arthroscopic control, typically using a probe or grasper introduced through the posterolateral working portal. Once anatomical reduction is confirmed, fixation is performed using suture anchors placed into the fibular head. Sutures are then passed through the attached soft tissues and avulsed fragment to allow stable reattachment of the arcuate complex.

**Figure 8:**
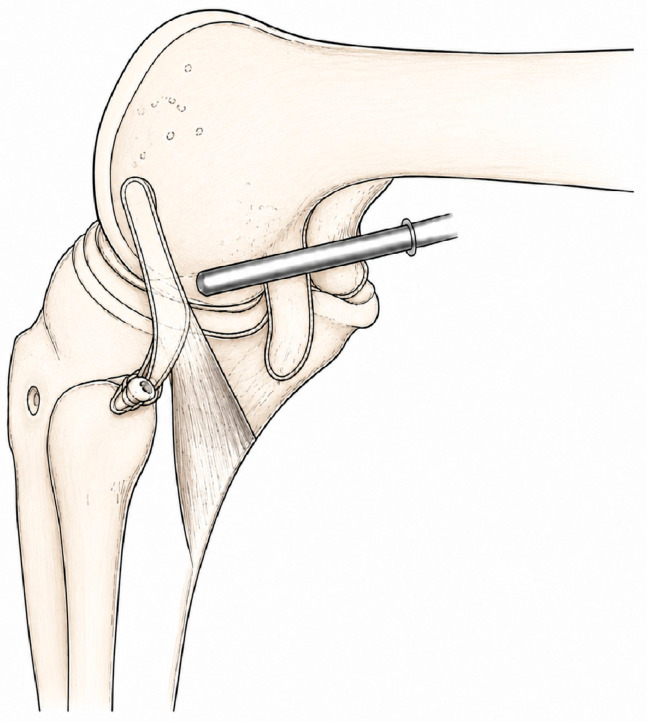
Schematic illustration of the final construct following all-arthroscopic repair of an arcuate complex avulsion fracture as described by Zhang et al. [25].

Final arthroscopic evaluation is performed to confirm fixation stability and restoration of the posterolateral structures (Figure 8). This technique enables anatomical repair of acute avulsion injuries while avoiding extensive soft-tissue dissection [25]. The authors reported satisfactory clinical outcomes in a single case involving a 45-year-old active patient who returned to daily and sports activities. At the 1-year follow-up, clinical examination demonstrated a 1+ posterior drawer test and external rotation 1° lower than the contralateral knee, while posterior stress radiographs obtained using the Telos Stress Device demonstrated a reduction in posterior tibial translation from 15.5 mm preoperatively to 6.3 mm postoperatively [25].

##### All-Arthroscopic Repair of Popliteus Tendon Avulsion (Salzler et al. [26])

The all-arthroscopic technique described by Salzler et al. is intended for the treatment of acute PT avulsion in the setting of multiligament knee injury and is indicated for acute tears in young, high-demand patients [26].

The procedure begins with standard diagnostic arthroscopy through anterolateral and anteromedial portals, allowing evaluation of associated intra-articular lesions. Additional posterolateral access is established to visualize the popliteus hiatus and the site of tendon avulsion at the femoral attachment. The avulsed PT is identified and mobilized, and the footprint at the lateral femoral condyle is prepared to promote healing.

Reduction of the tendon to its native femoral insertion is performed under arthroscopic visualization. Fixation is achieved using suture anchors placed at the anatomical footprint, with sutures passed through the tendon to secure stable reattachment. Arthroscopic control allows verification of appropriate tensioning and restoration of the PT’s anatomical course. The final assessment confirms the stability of the repair and restoration of the posterolateral structures. This technique enables anatomical restoration of the PT in acute injuries while avoiding extensive surgical exposure [26].

##### Arthroscopic Posterolateral Corner Stabilization with Popliteus Tenodesis (Hermanowicz et al. [27])

The arthroscopic technique described by Hermanowicz et al. aims to restore posterolateral stability through a popliteus-based tenodesis, providing a minimally invasive alternative to anatomic reconstruction in selected cases of PLC insufficiency [27].

The procedure begins with standard diagnostic arthroscopy through anterolateral and anteromedial portals, followed by assessment of the lateral compartment and popliteus region. Additional posterolateral visualization is established to identify the PT along its course from the musculotendinous junction to its femoral insertion.

After preparation of the lateral femoral condyle, the PT is mobilized and redirected to function as a static stabilizer. A fixation point is created at or near the femoral footprint of the LCL, and the tendon is secured using a suture anchor, thereby creating a tenodesis construct. This effectively reorients the PT to augment posterolateral stability, particularly against external rotation.

Final arthroscopic assessment is performed to confirm restoration of lateral compartment stability and appropriate tensioning of the construct. This technique avoids graft harvesting and extensive dissection, offering a less invasive option in selected patients [27].

## 4. Discussion

The most important finding of this narrative review is that arthroscopy represents a valuable option for both diagnosis and surgical management of PLC injuries. Rather than replacing MRI, arthroscopy should be regarded as a complementary diagnostic tool, particularly in patients with persistent clinical suspicion despite inconclusive imaging findings or in the setting of multiligament knee injuries. By providing direct visualization of the posterolateral compartment, dynamic assessment of joint stability, and identification of associated intra-articular lesions, arthroscopy may improve diagnostic accuracy, facilitate surgical planning, and support a more individualized treatment strategy. Although open techniques remain the most widely described and analyzed procedures, recent studies suggest that arthroscopic and arthroscopy-assisted reconstructions may achieve comparable clinical outcomes while potentially reducing surgical morbidity [13,18].

In their prospective study, Fahlbusch et al. reported that arthroscopic and open Arciero-based reconstruction techniques provided equivalent improvements in knee stability, as assessed by clinical stability testing and Rolimeter measurements of posterior tibial translation, as well as patient-reported outcome measures (Lysholm and IKDC scores), in chronic Fanelli type B injuries, without clinical failures or neurovascular complications [13]. Similarly, Weiss et al. demonstrated that arthroscopic PLC reconstruction based on Arciero and LaPrade principles restored posterolateral rotational instability, varus instability, and posterior drawer, as assessed by clinical stability testing and Rolimeter measurements, without major neurovascular complications [18].

One of the principal advantages of arthroscopy is the ability to simultaneously diagnose and treat associated intra-articular pathology, including anterior and posterior cruciate ligament injuries, meniscal tears, and chondral lesions. This integrated approach may facilitate comprehensive management of multiligament knee injuries during a single procedure, potentially improving surgical planning and treatment efficiency. This concept is supported by prospective clinical evidence showing that the simultaneous management of associated intra-articular lesions, including anterior cruciate ligament reconstruction and meniscal repair, is associated with favorable functional recovery and supports a comprehensive single-stage treatment strategy [38]. However, further comparative clinical studies are needed to determine whether this strategy translates into superior long-term functional outcomes [7,13,18].

An additional technical consideration concerns graft selection. The arthroscopic techniques described in the literature employ different graft options, including semitendinosus autograft; hamstring autograft; tibialis anterior allograft; and, more broadly in arthroscopic ligament reconstruction, peroneus longus autograft, which has shown encouraging clinical outcomes in selected patients [14,15,17,20,21,39]. Although no comparative evidence currently demonstrates the superiority of one graft over another specifically for arthroscopic PLC reconstruction, graft selection should be individualized according to patient characteristics, associated ligament injuries, graft availability, and surgeon preference. This concept is consistent with evidence from systematic reviews of ligament reconstruction, which support the use of different autograft options according to the clinical scenario rather than identifying a universally superior graft [37,40]. Autografts provide excellent biological incorporation but may be associated with donor-site morbidity, whereas allografts avoid graft harvest and may facilitate complex multiligament reconstructions, particularly when multiple grafts are required [7,8]. The successful use of ipsilateral hamstring tendons in other complex knee reconstructive procedures further supports their versatility as autograft options when appropriate [37,40].

The PLC is characterized by a complex anatomy and biomechanics and should be considered a functional unit in which the LCL, PT, PFL, posterolateral capsule, biceps femoris tendon, iliotibial band, and arcuate complex contribute synergistically to varus, rotational, and posterior stability [1,2,3,7]. This concept is clinically relevant because isolated single-structure reconstruction may fail to restore native knee kinematics when multiple stabilizers are involved. The LCL primarily resists varus stress, whereas the popliteus complex and PFL play a crucial role in controlling external tibial rotation and posterolateral rotatory instability [1,28]. Consequently, surgical strategies should be tailored according to the injured functional components.

Treatment selection remains dependent on injury severity, chronicity, tissue quality, associated lesions, and patient demands. Low-grade isolated PLC injuries may be treated conservatively, particularly grade I and selected isolated grade II lesions [3,5]. However, nonoperative management has shown poor outcomes in higher-grade instability, where persistent varus and rotational laxity may lead to chronic pain, functional impairment, altered joint loading, and progressive degenerative changes [3,5]. Surgical treatment is therefore generally recommended for grade III injuries, chronic symptomatic instability, combined ligament lesions, and high-demand patients with clinically significant grade II instability [5,7,30]. In these cases, the primary goal of treatment is restoration of the functional balance of the lateral and posterolateral stabilizers.

An important aspect highlighted in the literature is the need to recognize and address associated ligament injuries during the same surgical procedure. PLC injuries are rarely isolated and frequently occur in association with ACL, PCL, or multiligament knee injuries [4,5,7]. Failure to diagnose and treat PLC insufficiency may overload cruciate ligament grafts, resulting in persistent instability or graft failure [11,32]. This is particularly relevant in PCL-deficient knees, where posterolateral rotatory instability may be underestimated and isolated PCL reconstruction may be insufficient. In this setting, arthroscopy offers a major advantage over open procedures because it allows simultaneous evaluation and treatment of intra-articular lesions, including meniscal tears, chondral damage, cruciate ligament injuries, and PLC-related instability patterns within a single procedure.

The distinction between anatomical and non-anatomical reconstruction is another critical concept in PLC surgery. Non-anatomical procedures, including biceps rerouting, extra-articular tenodesis, and fibular sling techniques, may improve stability but do not accurately reproduce the native anatomy and biomechanics of the LCL, PT, and PFL [8,10]. Consequently, residual rotational instability or excessive constraint may persist, particularly in young and high-demand patients. In contrast, anatomical reconstruction aims to reproduce the native insertions and biomechanical function of the PLC stabilizers [19,22,23]. Recent systematic reviews have demonstrated lower failure rates for anatomical reconstruction compared with non-anatomical techniques in chronic grade III PLC injuries [8].

The choice between repair and reconstruction requires careful patient selection. Primary repair may be considered in selected acute lesions, particularly fibular head or femoral avulsion injuries, where anatomical reduction and fixation may restore native structures [25,26]. Repair of secondary stabilizers, including biceps femoris avulsions, lateral capsular lesions, and iliotibial band injuries, may further contribute to restoration of knee stability and biomechanics. However, midsubstance tears, chronic injuries, poor tissue quality, and grade II–III instability are generally more suitable for reconstruction procedures. White et al., in their systematic review, reported that isolated repair of acute PLC injuries may fail more frequently than reconstruction, with repair failure rates reported up to 40%, whereas reconstruction demonstrated lower failure rates [41]. Therefore, repair should not be considered a universal solution but rather a lesion-specific option reserved for selected repairable avulsion patterns.

The arthroscopic approach offers several theoretical and practical advantages compared with traditional open surgery. First, it improves visualization of anatomical landmarks that may be difficult to identify through open approaches, particularly the popliteus hiatus, posterolateral recess, PT course, and intra-articular landmarks used for tunnel placement [14,17,31,36,42]. Second, it allows dynamic assessment of lateral compartment insufficiency through findings such as the lateral gutter or lateral drive-through sign, which may reveal subtle or combined PLC instability [16,34,35]. Third, arthroscopic reconstruction may reduce soft-tissue trauma, scar formation, postoperative pain, infection risk, and overall surgical morbidity, potentially facilitating faster rehabilitation [6,13]. Finally, it may reduce surgical exposure and dissection around the common peroneal nerve [6,13].

Despite these potential advantages, arthroscopic PLC reconstruction remains technically demanding. A thorough understanding of arthroscopic posterolateral anatomy, portal placement, and transseptal visualization is mandatory to avoid complications. Concerns remain regarding tunnel malposition and neurovascular injury, particularly during drilling around the fibular head and posterior tibia [14,27]. Nevertheless, available studies have not reported major neurovascular complications when these procedures are performed by experienced arthroscopic knee surgeons [13,18]. Therefore, arthroscopic PLC reconstruction should currently be considered a promising but expertise-dependent approach reserved for surgeons experienced in advanced multiligament knee reconstruction and posterior compartment arthroscopy.

This review has limitations related both to its narrative design and to the currently available literature. Most studies investigating arthroscopic PLC surgery consist of technical notes, cadaveric investigations, small case series, or short-term comparative studies. High-quality randomized clinical trials are lacking, and substantial heterogeneity exists regarding injury patterns, classifications, associated cruciate ligament injuries, surgical techniques, rehabilitation protocols, and outcome measures. Furthermore, many studies combine PLC reconstruction with PCL or multiligament procedures, making it difficult to isolate the specific effect of arthroscopic PLC reconstruction. Future studies should also incorporate objective functional assessments, such as isokinetic muscle strength testing, alongside validated patient-reported outcome measures, to provide a more comprehensive evaluation of postoperative knee function [43]. Future comparative studies evaluating open, arthroscopic-assisted, and all-arthroscopic anatomical reconstructions should focus on standardized classifications, objective functional assessments, validated patient-reported outcome measures, complications, return-to-sport rates, and longer-term clinical outcomes.

## 5. Conclusions

Arthroscopy is a valuable option for diagnosing and surgically managing PLC injuries. Current evidence suggests that arthroscopic and arthroscopy-assisted anatomical reconstruction may restore knee stability and kinematics with outcomes comparable to those achieved with open techniques, while potentially reducing surgical morbidity and improving visualization of the posterolateral compartment. Nevertheless, the complexity of PLC anatomy and biomechanics requires careful patient selection, accurate identification and treatment of associated lesions, and restoration of the injured stabilizing structures through anatomical repair or reconstruction. Further high-quality studies are required to better define the indications, reproducibility, and long-term outcomes of arthroscopic PLC surgery.

## Figures and Tables

**Table 1:** Overview of the main arthroscopic and arthroscopy-assisted techniques for posterolateral corner (PLC) including indications, surgical concepts, advantages, and limitations.

Technique	Target Structures	Surgical Concept	Indications	Advantages	Limitations
Feng et al. [20]	PT	All-arthroscopic sling-type reconstruction	Selected cases of posterolateral rotatory instability with predominant popliteus involvement	Minimally invasive; restores popliteus function; avoids extensive soft-tissue dissection	Does not address the full PLC; limited applicability in combined or high-grade instability
Song et al. [21]	PFL	All-arthroscopic anatomical reconstruction	Selected cases with suspected PFL deficiency contributing to rotational instability	Targeted restoration of rotational stability; minimally invasive approach	Does not restore complete PLC stability; limited role in complex injuries
Ahn et al. [17]	LCL + popliteus complex	Arthroscopy-assisted anatomical reconstruction	High-grade PLC injuries and combined ligament instability	Anatomical reconstruction with improved visualization; reduced soft-tissue disruption compared to open techniques	Requires a mini-open approach; technically demanding
Frings et al. [14]	LCL + PT + PFL	All-arthroscopic multistructure reconstruction	Severe PLC instability and selected multiligament injuries	Comprehensive arthroscopic visualization; addresses multiple stabilizers	High technical complexity; limited reproducibility; steep learning curve
Liu et al. [15]	LCL + PT + PFL	All-arthroscopic anatomical reconstruction (biomechanical model)	Experimental application (cadaveric studies)	Demonstrates restoration of near-normal knee kinematics	No clinical validation; technically complex
Ohnishi et al. [24]	PT	Arthroscopic popliteus tendon reconstruction or stabilization	Selected cases of posterolateral rotatory instability with focal popliteus involvement	Minimally invasive; direct visualization of posterior compartment; targeted management of popliteus pathology	Does not restore all PLC stabilizers; technically demanding; limited clinical evidence
Zhang et al. [25]	Arcuate complex (fibular-based avulsion)	All-arthroscopic repair with suture anchors	Acute fibular head avulsion injuries with viable bony fragment	Anatomical repair preserving native structures; minimally invasive	Limited to acute avulsion injuries; not applicable in chronic or midsubstance tears
Salzler et al. [26]	PT	All-arthroscopic tendon repair	Acute popliteus tendon avulsion, often in multiligament injuries	Anatomical restoration; minimally invasive; allows concomitant procedures	Does not address complete PLC deficiency; limited role in chronic or complex instability
Hermanowicz et al. [27]	PT	Arthroscopic popliteus tenodesis	Selected cases of PLC insufficiency	No graft required; minimally invasive technique	Non-anatomic reconstruction; limited biomechanical restoration

Abbreviations: PLC = posterolateral corner; PT = popliteus tendon; PFL = popliteofibular ligament; LCL = lateral collateral ligament.

## Data Availability

The data presented in this study are available in the article.

## Abbreviations

The following abbreviations are used in this manuscript:

ACL	Anterior cruciate ligament
ALL	Anterolateral ligament
LCL	Lateral collateral ligament
LM	Lateral meniscus
MRI	Magnetic resonance imaging
PCL	Posterior cruciate ligament
PFL	Popliteofibular ligament
PLC	Posterolateral corner
PLRI	Posterolateral rotatory instability
PT	Popliteus tendon
AL	Anterolateral
AM	Anteromedial
LTC	Lateral tibial condyle
PM	Posteromedial
PL	Posterolateral
FF	Fabellofibular ligament
BF	Biceps femoris
PMM	Popliteus muscle
